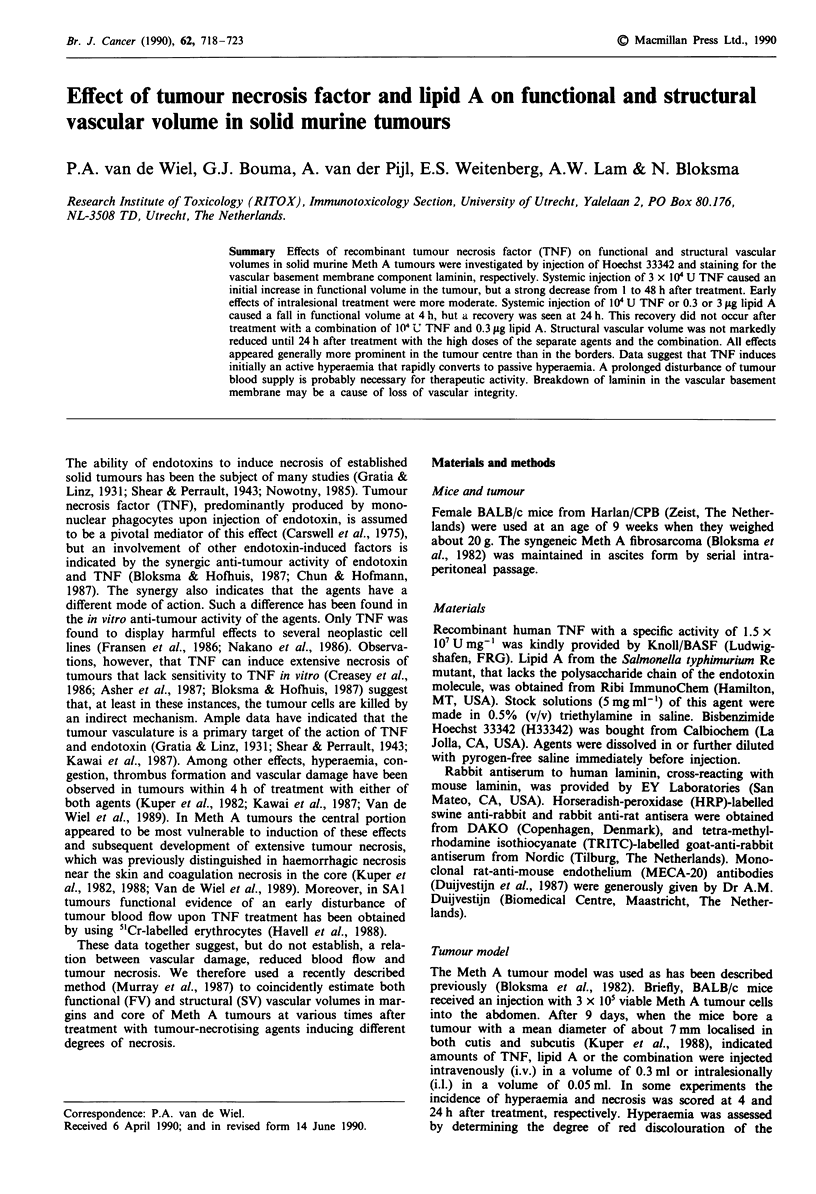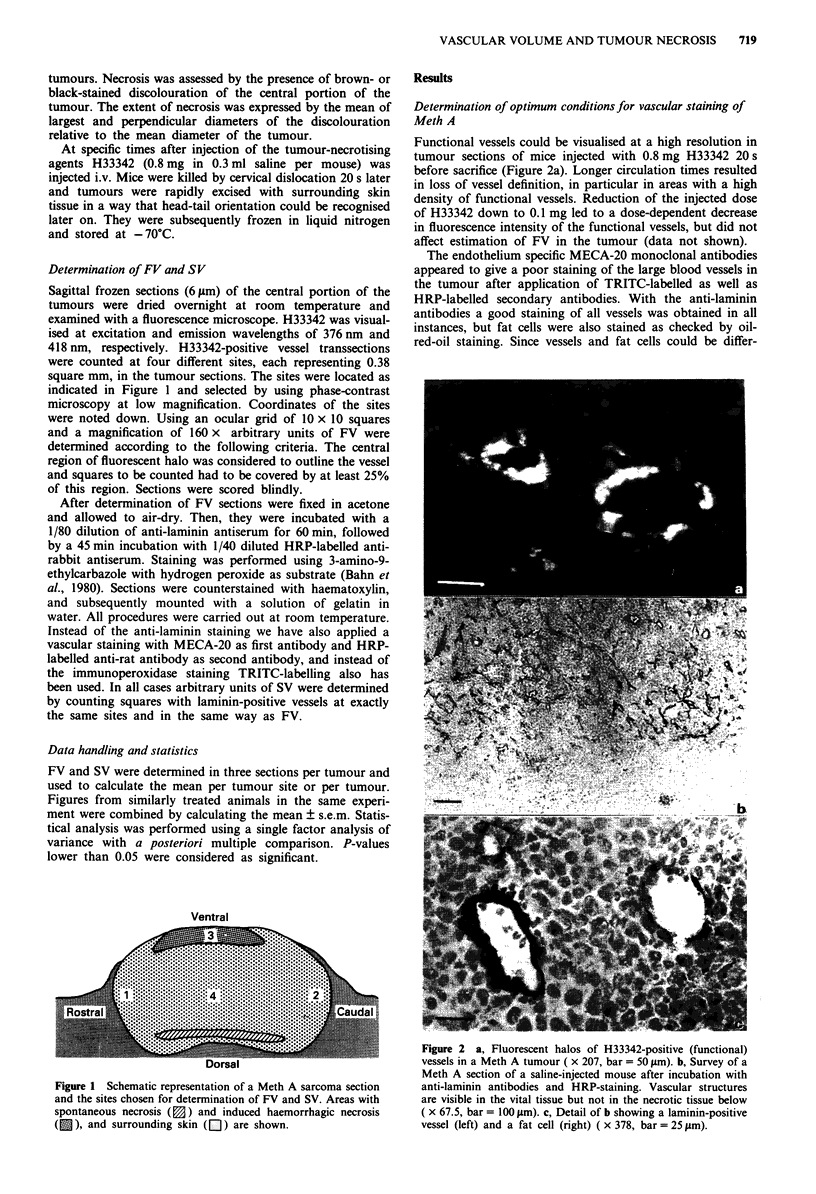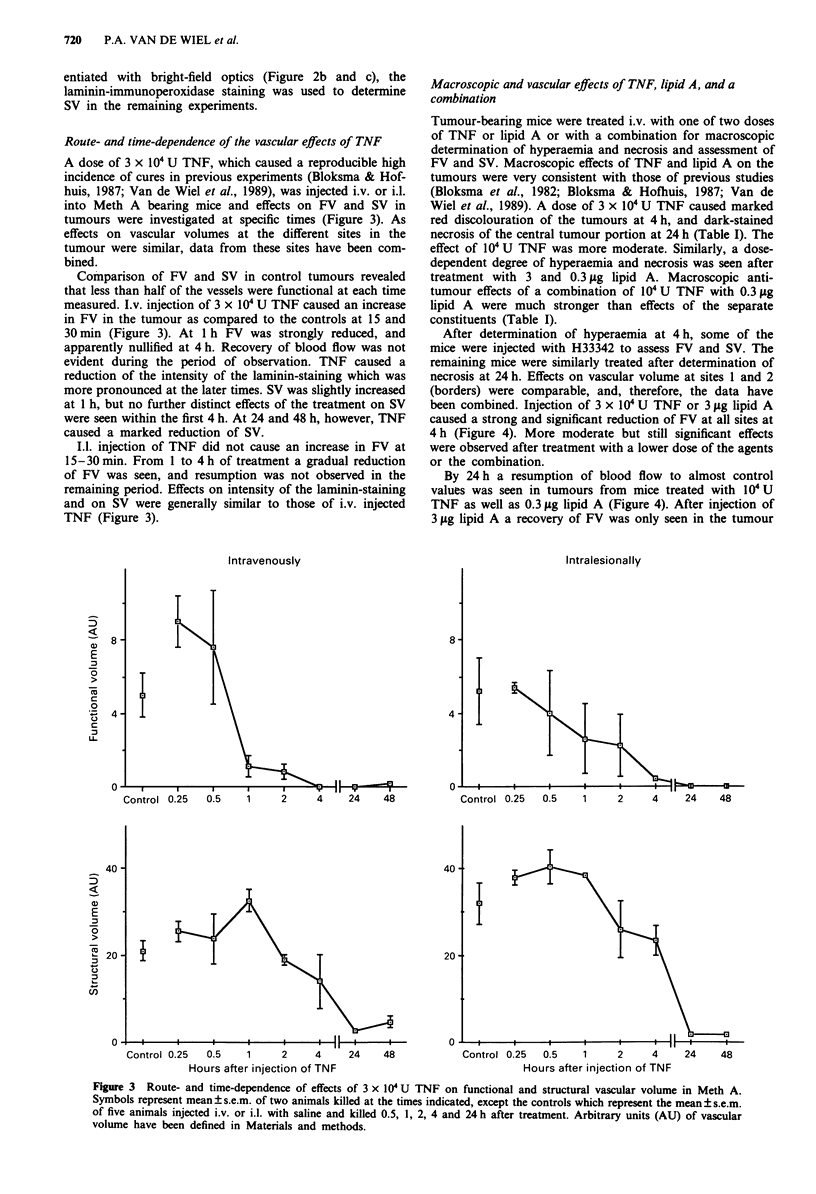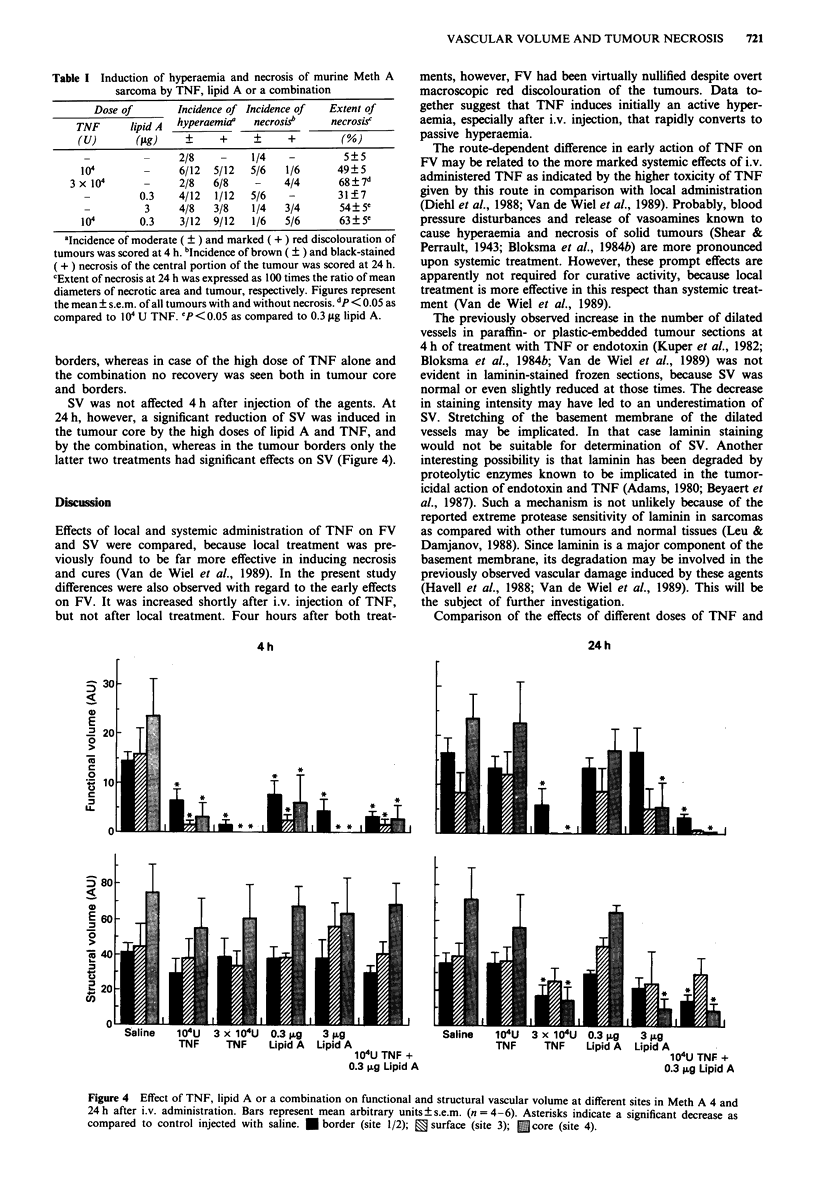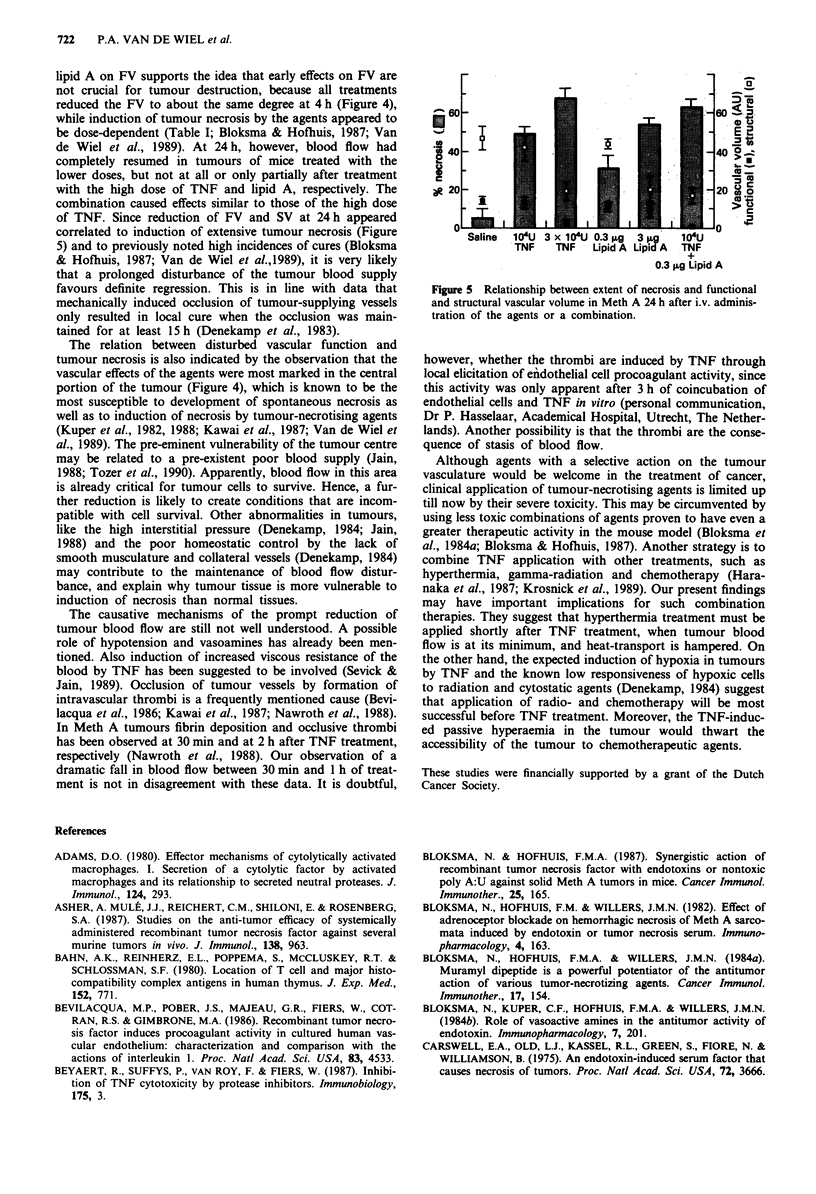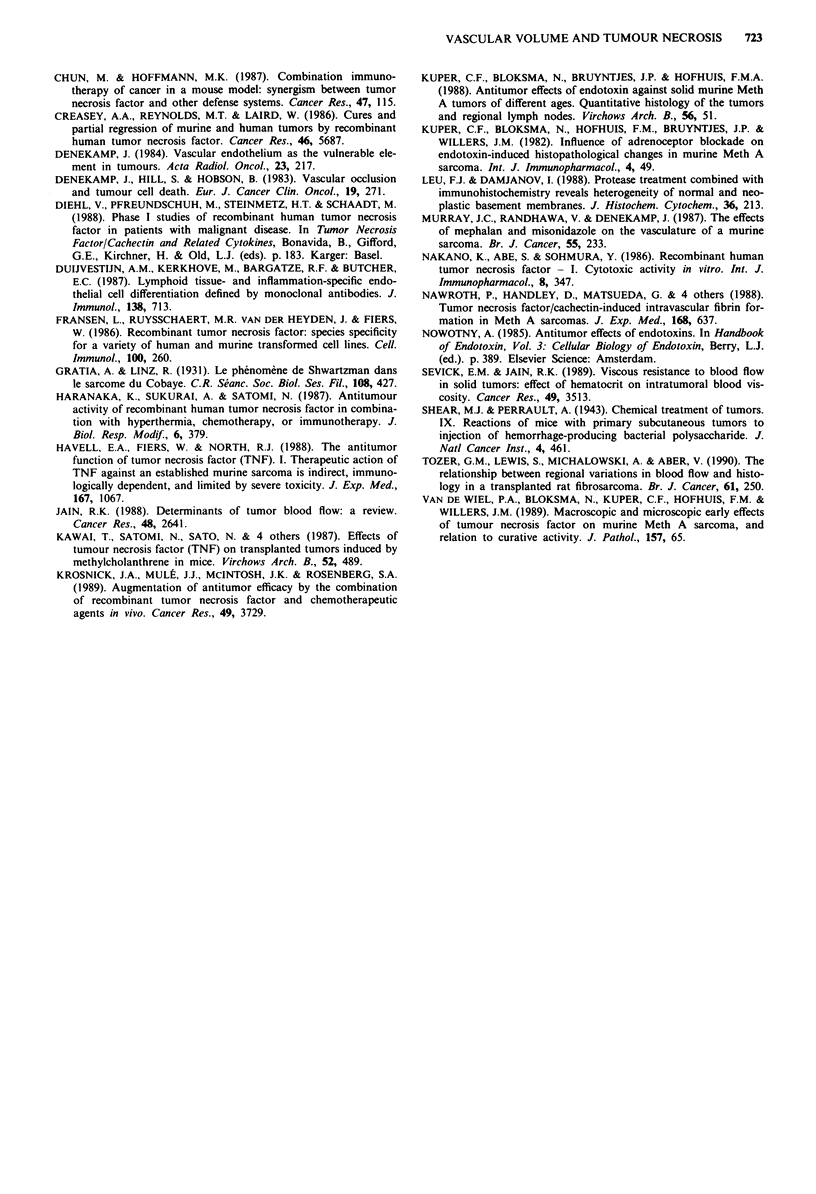# Effect of tumour necrosis factor and lipid A on functional and structural vascular volume in solid murine tumours.

**DOI:** 10.1038/bjc.1990.366

**Published:** 1990-11

**Authors:** P. A. van de Weil, G. J. Bouma, A. van der Pijl, E. S. Weitenberg, A. W. Lam, N. Bloksma

**Affiliations:** Research Institute of Toxicology (RITOX), University of Utrecht, The Netherlands.

## Abstract

**Images:**


 © Macmillan Press Ltd., 1990

### Effect of tumour necrosis factor and lipid A on functional and structural vascular volume in solid murine tumours

P.A. van de Wiel, G.J. Bouma, A. van der Pijl, E.S. Weitenberg, A.W. Lam & N. Bloksma

*Research Institute of Toxicology (RITOX), Immunotoxicology Section, University of Utrecht, Yalelaan 2, PO Box 80.176, NL-3508 TD, Utrecht, The Netherlands.*

**Summary** Effects of recombinant tumour necrosis factor (TNF) on functional and structural vascular volumes in solid murine Meth A tumours were investigated by injection of Hoechst 33342 and staining for the vascular basement membrane component laminin, respectively. Systemic injection of $3 \times 10^4$ U TNF caused an initial increase in functional volume in the tumour, but a strong decrease from 1 to 48 h after treatment. Early effects of intralesional treatment were more moderate. Systemic injection of $10^4$ U TNF or 0.3 or 3 µg lipid A caused a fall in functional volume at 4 h, but a recovery was seen at 24 h. This recovery did not occur after treatment with a combination of $10^4$ U TNF and 0.3 µg lipid A. Structural vascular volume was not markedly reduced until 24 h after treatment with the high doses of the separate agents and the combination. All effects appeared generally more prominent in the tumour centre than in the borders. Data suggest that TNF induces initially an active hyperaemia that rapidly converts to passive hyperaemia. A prolonged disturbance of tumour blood supply is probably necessary for therapeutic activity. Breakdown of laminin in the vascular basement membrane may be a cause of loss of vascular integrity.

The ability of endotoxins to induce necrosis of established solid tumours has been the subject of many studies (Gratia & Linz, 1931; Shear & Perrault, 1943; Nowotny, 1985). Tumour necrosis factor (TNF), predominantly produced by mononuclear phagocytes upon injection of endotoxin, is assumed to be a pivotal mediator of this effect (Carswell *et al.*, 1975), but an involvement of other endotoxin-induced factors is indicated by the synergic anti-tumour activity of endotoxin and TNF (Bloksma & Hofhuis, 1987; Chun & Hofmann, 1987). The synergy also indicates that the agents have a different mode of action. Such a difference has been found in the *in vitro* anti-tumour activity of the agents. Only TNF was found to display harmful effects to several neoplastic cell lines (Fransen *et al.*, 1986; Nakano *et al.*, 1986). Observations, however, that TNF can induce extensive necrosis of tumours that lack sensitivity to TNF *in vitro* (Creasey *et al.*, 1986; Asher *et al.*, 1987; Bloksma & Hofhuis, 1987) suggest that, at least in these instances, the tumour cells are killed by an indirect mechanism. Ample data have indicated that the tumour vasculature is a primary target of the action of TNF and endotoxin (Gratia & Linz, 1931; Shear & Perrault, 1943; Kawai *et al.*, 1987). Among other effects, hyperaemia, congestion, thrombus formation and vascular damage have been observed in tumours within 4 h of treatment with either of both agents (Kuper *et al.*, 1982; Kawai *et al.*, 1987; Van de Wiel *et al.*, 1989). In Meth A tumours the central portion appeared to be most vulnerable to induction of these effects and subsequent development of extensive tumour necrosis, which was previously distinguished in haemorrhagic necrosis near the skin and coagulation necrosis in the core (Kuper *et al.*, 1982, 1988; Van de Wiel *et al.*, 1989). Moreover, in SA1 tumours functional evidence of an early disturbance of tumour blood flow upon TNF treatment has been obtained by using $^{51}$Cr-labelled erythrocytes (Havell *et al.*, 1988).

These data together suggest, but do not establish, a relation between vascular damage, reduced blood flow and tumour necrosis. We therefore used a recently described method (Murray *et al.*, 1987) to coincidently estimate both functional (FV) and structural (SV) vascular volumes in margins and core of Meth A tumours at various times after treatment with tumour-necrotising agents inducing different degrees of necrosis.

Correspondence: P.A. van de Wiel.
Received 6 April 1990; and in revised form 14 June 1990.

### Materials and methods

### *Mice and tumour*

Female BALB/c mice from Harlan/CPB (Zeist, The Netherlands) were used at an age of 9 weeks when they weighed about 20 g. The syngeneic Meth A fibrosarcoma (Bloksma *et al.*, 1982) was maintained in ascites form by serial intraperitoneal passage.

### *Materials*

Recombinant human TNF with a specific activity of $1.5 \times 10^7$ U mg$^{-1}$ was kindly provided by Knoll/BASF (Ludwigshafen, FRG). Lipid A from the *Salmonella typhimurium* Re mutant, that lacks the polysaccharide chain of the endotoxin molecule, was obtained from Ribi ImmunoChem (Hamilton, MT, USA). Stock solutions (5 mg ml$^{-1}$) of this agent were made in 0.5% (v/v) triethylamine in saline. Bisbenzimide Hoechst 33342 (H33342) was bought from Calbiochem (La Jolla, CA, USA). Agents were dissolved in or further diluted with pyrogen-free saline immediately before injection.

Rabbit antiserum to human laminin, cross-reacting with mouse laminin, was provided by EY Laboratories (San Mateo, CA, USA). Horseradish-peroxidase (HRP)-labelled swine anti-rabbit and rabbit anti-rat antisera were obtained from DAKO (Copenhagen, Denmark), and tetra-methylrhodamine isothiocyanate (TRITC)-labelled goat-anti-rabbit antiserum from Nordic (Tilburg, The Netherlands). Monoclonal rat-anti-mouse endothelium (MECA-20) antibodies (Duijvestijn *et al.*, 1987) were generously given by Dr A.M. Duijvestijn (Biomedical Centre, Maastricht, The Netherlands).

### *Tumour model*

The Meth A tumour model was used as has been described previously (Bloksma *et al.*, 1982). Briefly, BALB/c mice received an injection with $3 \times 10^5$ viable Meth A tumour cells into the abdomen. After 9 days, when the mice bore a tumour with a mean diameter of about 7 mm localised in both cutis and subcutis (Kuper *et al.*, 1988), indicated amounts of TNF, lipid A or the combination were injected intravenously (i.v.) in a volume of 0.3 ml or intralesionally (i.l.) in a volume of 0.05 ml. In some experiments the incidence of hyperaemia and necrosis was scored at 4 and 24 h after treatment, respectively. Hyperaemia was assessed by determining the degree of red discolouration of the tumours. Necrosis was assessed by the presence of brown- or black-stained discolouration of the central portion of the tumour. The extent of necrosis was expressed by the mean of largest and perpendicular diameters of the discolouration relative to the mean diameter of the tumour.

At specific times after injection of the tumour-necrotising agents H33342 (0.8 mg in 0.3 ml saline per mouse) was injected i.v. Mice were killed by cervical dislocation 20 s later and tumours were rapidly excised with surrounding skin tissue in a way that head-tail orientation could be recognised later on. They were subsequently frozen in liquid nitrogen and stored at $-70°C$.

### *Determination of FV and SV*

Sagittal frozen sections (6 $\mu m$) of the central portion of the tumours were dried overnight at room temperature and examined with a fluorescence microscope. H33342 was visualised at excitation and emission wavelengths of 376 nm and 418 nm, respectively. H33342-positive vessel transsections were counted at four different sites, each representing 0.38 square mm, in the tumour sections. The sites were located as indicated in Figure 1 and selected by using phase-contrast microscopy at low magnification. Coordinates of the sites were noted down. Using an ocular grid of $10 \times 10$ squares and a magnification of $160 \times$ arbitrary units of FV were determined according to the following criteria. The central region of fluorescent halo was considered to outline the vessel and squares to be counted had to be covered by at least 25% of this region. Sections were scored blindly.

After determination of FV sections were fixed in acetone and allowed to air-dry. Then, they were incubated with a 1/80 dilution of anti-laminin antiserum for 60 min, followed by a 45 min incubation with 1/40 diluted HRP-labelled anti-rabbit antiserum. Staining was performed using 3-amino-9-ethylcarbazole with hydrogen peroxide as substrate (Bahn *et al.*, 1980). Sections were counterstained with haematoxylin, and subsequently mounted with a solution of gelatin in water. All procedures were carried out at room temperature. Instead of the anti-laminin staining we have also applied a vascular staining with MECA-20 as first antibody and HRP-labelled anti-rat antibody as second antibody, and instead of the immunoperoxidase staining TRITC-labelling also has been used. In all cases arbitrary units of SV were determined by counting squares with laminin-positive vessels at exactly the same sites and in the same way as FV.

### *Data handling and statistics*

FV and SV were determined in three sections per tumour and used to calculate the mean per tumour site or per tumour. Figures from similarly treated animals in the same experiment were combined by calculating the mean ± s.e.m. Statistical analysis was performed using a single factor analysis of variance with *a posteriori* multiple comparison. *P*-values lower than 0.05 were considered as significant.

**Figure 1** Schematic representation of a Meth A sarcoma section and the sites chosen for determination of FV and SV. Areas with spontaneous necrosis (▨) and induced haemorrhagic necrosis (▩), and surrounding skin (□) are shown.

### Results

### *Determination of optimum conditions for vascular staining of Meth A*

Functional vessels could be visualised at a high resolution in tumour sections of mice injected with 0.8 mg H33342 20 s before sacrifice (Figure 2a). Longer circulation times resulted in loss of vessel definition, in particular in areas with a high density of functional vessels. Reduction of the injected dose of H33342 down to 0.1 mg led to a dose-dependent decrease in fluorescence intensity of the functional vessels, but did not affect estimation of FV in the tumour (data not shown).

The endothelium specific MECA-20 monoclonal antibodies appeared to give a poor staining of the large blood vessels in the tumour after application of TRITC-labelled as well as HRP-labelled secondary antibodies. With the anti-laminin antibodies a good staining of all vessels was obtained in all instances, but fat cells were also stained as checked by oil-red-oil staining. Since vessels and fat cells could be differ-

**Figure 2** **a**, Fluorescent halos of H33342-positive (functional) vessels in a Meth A tumour ( $\times$ 207, bar = 50 $\mu m$). **b**, Survey of a Meth A section of a saline-injected mouse after incubation with anti-laminin antibodies and HRP-staining. Vascular structures are visible in the vital tissue but not in the necrotic tissue below ( $\times$ 67.5, bar = 100 $\mu m$). **c**, Detail of **b** showing a laminin-positive vessel (left) and a fat cell (right) ( $\times$ 378, bar = 25 $\mu m$).

entiated with bright-field optics (Figure 2b and c), the laminin-immunoperoxidase staining was used to determine SV in the remaining experiments.

*Route- and time-dependence of the vascular effects of TNF*

A dose of $3 \times 10^4$ U TNF, which caused a reproducible high incidence of cures in previous experiments (Bloksma & Hofhuis, 1987; Van de Wiel *et al.*, 1989), was injected i.v. or i.l. into Meth A bearing mice and effects on FV and SV in tumours were investigated at specific times (Figure 3). As effects on vascular volumes at the different sites in the tumour were similar, data from these sites have been combined.

Comparison of FV and SV in control tumours revealed that less than half of the vessels were functional at each time measured. I.v. injection of $3 \times 10^4$ U TNF caused an increase in FV in the tumour as compared to the controls at 15 and 30 min (Figure 3). At 1 h FV was strongly reduced, and apparently nullified at 4 h. Recovery of blood flow was not evident during the period of observation. TNF caused a reduction of the intensity of the laminin-staining which was more pronounced at the later times. SV was slightly increased at 1 h, but no further distinct effects of the treatment on SV were seen within the first 4 h. At 24 and 48 h, however, TNF caused a marked reduction of SV.

I.l. injection of TNF did not cause an increase in FV at 15–30 min. From 1 to 4 h of treatment a gradual reduction of FV was seen, and resumption was not observed in the remaining period. Effects on intensity of the laminin-staining and on SV were generally similar to those of i.v. injected TNF (Figure 3).

*Macroscopic and vascular effects of TNF, lipid A, and a combination*

Tumour-bearing mice were treated i.v. with one of two doses of TNF or lipid A or with a combination for macroscopic determination of hyperaemia and necrosis and assessment of FV and SV. Macroscopic effects of TNF and lipid A on the tumours were very consistent with those of previous studies (Bloksma *et al.*, 1982; Bloksma & Hofhuis, 1987; Van de Wiel *et al.*, 1989). A dose of $3 \times 10^4$ U TNF caused marked red discolouration of the tumours at 4 h, and dark-stained necrosis of the central tumour portion at 24 h (Table I). The effect of $10^4$ U TNF was more moderate. Similarly, a dose-dependent degree of hyperaemia and necrosis was seen after treatment with 3 and 0.3 µg lipid A. Macroscopic anti-tumour effects of a combination of $10^4$ U TNF with 0.3 µg lipid A were much stronger than effects of the separate constituents (Table I).

After determination of hyperaemia at 4 h, some of the mice were injected with H33342 to assess FV and SV. The remaining mice were similarly treated after determination of necrosis at 24 h. Effects on vascular volume at sites 1 and 2 (borders) were comparable, and, therefore, the data have been combined. Injection of $3 \times 10^4$ U TNF or 3 µg lipid A caused a strong and significant reduction of FV at all sites at 4 h (Figure 4). More moderate but still significant effects were observed after treatment with a lower dose of the agents or the combination.

By 24 h a resumption of blood flow to almost control values was seen in tumours from mice treated with $10^4$ U TNF as well as 0.3 µg lipid A (Figure 4). After injection of 3 µg lipid A a recovery of FV was only seen in the tumour

**Figure 3** Route- and time-dependence of effects of $3 \times 10^4$ U TNF on functional and structural vascular volume in Meth A. Symbols represent mean ± s.e.m. of two animals killed at the times indicated, except the controls which represent the mean ± s.e.m. of five animals injected i.v. or i.l. with saline and killed 0.5, 1, 2, 4 and 24 h after treatment. Arbitrary units (AU) of vascular volume have been defined in Materials and methods.

**Table I** Induction of hyperaemia and necrosis of murine Meth A sarcoma by TNF, lipid A or a combination

| *Dose of* | | *Incidence of hyperaemia*[a] | | *Incidence of necrosis*[b] | | *Extent of necrosis*[c] |
|---|---|---|---|---|---|---|
| *TNF (U)* | *lipid A (μg)* | ± | + | ± | + | *(%)* |
| – | – | 2/8 | – | 1/4 | – | 5±5 |
| $10^4$ | – | 6/12 | 5/12 | 5/6 | 1/6 | 49±5 |
| $3 \times 10^4$ | – | 2/8 | 6/8 | – | 4/4 | 68±7[d] |
| – | 0.3 | 4/12 | 1/12 | 5/6 | – | 31±7 |
| – | 3 | 4/8 | 3/8 | 1/4 | 3/4 | 54±5[e] |
| $10^4$ | 0.3 | 3/12 | 9/12 | 1/6 | 5/6 | 63±5[e] |

[a]Incidence of moderate ( ± ) and marked ( + ) red discolouration of tumours was scored at 4 h. [b]Incidence of brown ( ± ) and black-stained ( + ) necrosis of the central portion of the tumour was scored at 24 h. [c]Extent of necrosis at 24 h was expressed as 100 times the ratio of mean diameters of necrotic area and tumour, respectively. Figures represent the mean ± s.e.m. of all tumours with and without necrosis. [d]$P < 0.05$ as compared to $10^4$ U TNF. [e]$P < 0.05$ as compared to 0.3 μg lipid A.

borders, whereas in case of the high dose of TNF alone and the combination no recovery was seen both in tumour core and borders.

SV was not affected 4 h after injection of the agents. At 24 h, however, a significant reduction of SV was induced in the tumour core by the high doses of lipid A and TNF, and by the combination, whereas in the tumour borders only the latter two treatments had significant effects on SV (Figure 4).

### Discussion

Effects of local and systemic administration of TNF on FV and SV were compared, because local treatment was previously found to be far more effective in inducing necrosis and cures (Van de Wiel *et al.*, 1989). In the present study differences were also observed with regard to the early effects on FV. It was increased shortly after i.v. injection of TNF, but not after local treatment. Four hours after both treatments, however, FV had been virtually nullified despite overt macroscopic red discolouration of the tumours. Data together suggest that TNF induces initially an active hyperaemia, especially after i.v. injection, that rapidly converts to passive hyperaemia.

The route-dependent difference in early action of TNF on FV may be related to the more marked systemic effects of i.v. administered TNF as indicated by the higher toxicity of TNF given by this route in comparison with local administration (Diehl *et al.*, 1988; Van de Wiel *et al.*, 1989). Probably, blood pressure disturbances and release of vasoamines known to cause hyperaemia and necrosis of solid tumours (Shear & Perrault, 1943; Bloksma *et al.*, 1984*b*) are more pronounced upon systemic treatment. However, these prompt effects are apparently not required for curative activity, because local treatment is more effective in this respect than systemic treatment (Van de Wiel *et al.*, 1989).

The previously observed increase in the number of dilated vessels in paraffin- or plastic-embedded tumour sections at 4 h of treatment with TNF or endotoxin (Kuper *et al.*, 1982; Bloksma *et al.*, 1984*b*; Van de Wiel *et al.*, 1989) was not evident in laminin-stained frozen sections, because SV was normal or even slightly reduced at those times. The decrease in staining intensity may have led to an underestimation of SV. Stretching of the basement membrane of the dilated vessels may be implicated. In that case laminin staining would not be suitable for determination of SV. Another interesting possibility is that laminin has been degraded by proteolytic enzymes known to be implicated in the tumoricidal action of endotoxin and TNF (Adams, 1980; Beyaert *et al.*, 1987). Such a mechanism is not unlikely because of the reported extreme protease sensitivity of laminin in sarcomas as compared with other tumours and normal tissues (Leu & Damjanov, 1988). Since laminin is a major component of the basement membrane, its degradation may be involved in the previously observed vascular damage induced by these agents (Havell *et al.*, 1988; Van de Wiel *et al.*, 1989). This will be the subject of further investigation.

Comparison of the effects of different doses of TNF and

**Figure 4** Effect of TNF, lipid A or a combination on functional and structural vascular volume at different sites in Meth A 4 and 24 h after i.v. administration. Bars represent mean arbitrary units ± s.e.m. ($n = 4–6$). Asterisks indicate a significant decrease as compared to control injected with saline. ■ border (site 1/2); ▨ surface (site 3); ▩ core (site 4).

lipid A on FV supports the idea that early effects on FV are not crucial for tumour destruction, because all treatments reduced the FV to about the same degree at 4 h (Figure 4), while induction of tumour necrosis by the agents appeared to be dose-dependent (Table I; Bloksma & Hofhuis, 1987; Van de Wiel *et al.*, 1989). At 24 h, however, blood flow had completely resumed in tumours of mice treated with the lower doses, but not at all or only partially after treatment with the high dose of TNF and lipid A, respectively. The combination caused effects similar to those of the high dose of TNF. Since reduction of FV and SV at 24 h appeared correlated to induction of extensive tumour necrosis (Figure 5) and to previously noted high incidences of cures (Bloksma & Hofhuis, 1987; Van de Wiel *et al.*,1989), it is very likely that a prolonged disturbance of the tumour blood supply favours definite regression. This is in line with data that mechanically induced occlusion of tumour-supplying vessels only resulted in local cure when the occlusion was maintained for at least 15 h (Denekamp *et al.*, 1983).

The relation between disturbed vascular function and tumour necrosis is also indicated by the observation that the vascular effects of the agents were most marked in the central portion of the tumour (Figure 4), which is known to be the most susceptible to development of spontaneous necrosis as well as to induction of necrosis by tumour-necrotising agents (Kuper *et al.*, 1982, 1988; Kawai *et al.*, 1987; Van de Wiel *et al.*, 1989). The pre-eminent vulnerability of the tumour centre may be related to a pre-existent poor blood supply (Jain, 1988; Tozer *et al.*, 1990). Apparently, blood flow in this area is already critical for tumour cells to survive. Hence, a further reduction is likely to create conditions that are incompatible with cell survival. Other abnormalities in tumours, like the high interstitial pressure (Denekamp, 1984; Jain, 1988) and the poor homeostatic control by the lack of smooth musculature and collateral vessels (Denekamp, 1984) may contribute to the maintenance of blood flow disturbance, and explain why tumour tissue is more vulnerable to induction of necrosis than normal tissues.

The causative mechanisms of the prompt reduction of tumour blood flow are still not well understood. A possible role of hypotension and vasoamines has already been mentioned. Also induction of increased viscous resistance of the blood by TNF has been suggested to be involved (Sevick & Jain, 1989). Occlusion of tumour vessels by formation of intravascular thrombi is a frequently mentioned cause (Bevilacqua *et al.*, 1986; Kawai *et al.*, 1987; Nawroth *et al.*, 1988). In Meth A tumours fibrin deposition and occlusive thrombi has been observed at 30 min and at 2 h after TNF treatment, respectively (Nawroth *et al.*, 1988). Our observation of a dramatic fall in blood flow between 30 min and 1 h of treatment is not in disagreement with these data. It is doubtful, however, whether the thrombi are induced by TNF through local elicitation of endothelial cell procoagulant activity, since this activity was only apparent after 3 h of coincubation of endothelial cells and TNF *in vitro* (personal communication, Dr P. Hasselaar, Academical Hospital, Utrecht, The Netherlands). Another possibility is that the thrombi are the consequence of stasis of blood flow.

**Figure 5** Relationship between extent of necrosis and functional and structural vascular volume in Meth A 24 h after i.v. administration of the agents or a combination.

Although agents with a selective action on the tumour vasculature would be welcome in the treatment of cancer, clinical application of tumour-necrotising agents is limited up till now by their severe toxicity. This may be circumvented by using less toxic combinations of agents proven to have even a greater therapeutic activity in the mouse model (Bloksma *et al.*, 1984*a*; Bloksma & Hofhuis, 1987). Another strategy is to combine TNF application with other treatments, such as hyperthermia, gamma-radiation and chemotherapy (Haranaka *et al.*, 1987; Krosnick *et al.*, 1989). Our present findings may have important implications for such combination therapies. They suggest that hyperthermia treatment must be applied shortly after TNF treatment, when tumour blood flow is at its minimum, and heat-transport is hampered. On the other hand, the expected induction of hypoxia in tumours by TNF and the known low responsiveness of hypoxic cells to radiation and cytostatic agents (Denekamp, 1984) suggest that application of radio- and chemotherapy will be most successful before TNF treatment. Moreover, the TNF-induced passive hyperaemia in the tumour would thwart the accessibility of the tumour to chemotherapeutic agents.

These studies were financially supported by a grant of the Dutch Cancer Society.

### References

ADAMS, D.O. (1980). Effector mechanisms of cytolytically activated macrophages. I. Secretion of a cytolytic factor by activated macrophages and its relationship to secreted neutral proteases. *J. Immunol.*, **124**, 293.

ASHER, A. MULÉ, J.J., REICHERT, C.M., SHILONI, E. & ROSENBERG, S.A. (1987). Studies on the anti-tumor efficacy of systemically administered recombinant tumor necrosis factor against several murine tumors *in vivo*. *J. Immunol.*, **138**, 963.

BAHN, A.K., REINHERZ, E.L., POPPEMA, S., McCLUSKEY, R.T. & SCHLOSSMAN, S.F. (1980). Location of T cell and major histocompatibility complex antigens in human thymus. *J. Exp. Med.*, **152**, 771.

BEVILACQUA, M.P., POBER, J.S., MAJEAU, G.R., FIERS, W., COTRAN, R.S. & GIMBRONE, M.A. (1986). Recombinant tumor necrosis factor induces procoagulant activity in cultured human vascular endothelium: characterization and comparison with the actions of interleukin 1. *Proc. Natl Acad. Sci. USA*, **83**, 4533.

BEYAERT, R., SUFFYS, P., VAN ROY, F. & FIERS, W. (1987). Inhibition of TNF cytotoxicity by protease inhibitors. *Immunobiology*, **175**, 3.

BLOKSMA, N. & HOFHUIS, F.M.A. (1987). Synergistic action of recombinant tumor necrosis factor with endotoxins or nontoxic poly A:U against solid Meth A tumors in mice. *Cancer Immunol. Immunother.*, **25**, 165.

BLOKSMA, N., HOFHUIS, F.M. & WILLERS, J.M.N. (1982). Effect of adrenoceptor blockade on hemorrhagic necrosis of Meth A sarcomata induced by endotoxin or tumor necrosis serum. *Immunopharmacology*, **4**, 163.

BLOKSMA, N., HOFHUIS, F.M.A. & WILLERS, J.M.N. (1984*a*). Muramyl dipeptide is a powerful potentiator of the antitumor action of various tumor-necrotizing agents. *Cancer Immunol. Immunother.*, **17**, 154.

BLOKSMA, N., KUPER, C.F., HOFHUIS, F.M.A. & WILLERS, J.M.N. (1984*b*). Role of vasoactive amines in the antitumor activity of endotoxin. *Immunopharmacology*, **7**, 201.

CARSWELL, E.A., OLD, L.J., KASSEL, R.L., GREEN, S., FIORE, N. & WILLIAMSON, B. (1975). An endotoxin-induced serum factor that causes necrosis of tumors. *Proc. Natl Acad. Sci. USA*, **72**, 3666.

CHUN, M. & HOFFMANN, M.K. (1987). Combination immunotherapy of cancer in a mouse model: synergism between tumor necrosis factor and other defense systems. *Cancer Res.*, **47**, 115.

CREASEY, A.A., REYNOLDS, M.T. & LAIRD, W. (1986). Cures and partial regression of murine and human tumors by recombinant human tumor necrosis factor. *Cancer Res.*, **46**, 5687.

DENEKAMP, J. (1984). Vascular endothelium as the vulnerable element in tumours. *Acta Radiol. Oncol.*, **23**, 217.

DENEKAMP, J., HILL, S. & HOBSON, B. (1983). Vascular occlusion and tumour cell death. *Eur. J. Cancer Clin. Oncol.*, **19**, 271.

DIEHL, V., PFREUNDSCHUH, M., STEINMETZ, H.T. & SCHAADT, M. (1988). Phase I studies of recombinant human tumor necrosis factor in patients with malignant disease. In *Tumor Necrosis Factor/Cachectin and Related Cytokines*, Bonavida, B., Gifford, G.E., Kirchner, H. & Old, L.J. (eds). p. 183. Karger: Basel.

DUIJVESTIJN, A.M., KERKHOVE, M., BARGATZE, R.F. & BUTCHER, E.C. (1987). Lymphoid tissue- and inflammation-specific endothelial cell differentiation defined by monoclonal antibodies. *J. Immunol.*, **138**, 713.

FRANSEN, L., RUYSSCHAERT, M.R. VAN DER HEYDEN, J. & FIERS, W. (1986). Recombinant tumor necrosis factor: species specificity for a variety of human and murine transformed cell lines. *Cell. Immunol.*, **100**, 260.

GRATIA, A. & LINZ, R. (1931). Le phénomène de Shwartzman dans le sarcome du Cobaye. *C.R. Séanc. Soc. Biol. Ses. Fil.*, **108**, 427.

HARANAKA, K., SUKURAI, A. & SATOMI, N. (1987). Antitumour activity of recombinant human tumor necrosis factor in combination with hyperthermia, chemotherapy, or immunotherapy. *J. Biol. Resp. Modif.*, **6**, 379.

HAVELL, E.A., FIERS, W. & NORTH, R.J. (1988). The antitumor function of tumor necrosis factor (TNF). I. Therapeutic action of TNF against an established murine sarcoma is indirect, immunologically dependent, and limited by severe toxicity. *J. Exp. Med.*, **167**, 1067.

JAIN, R.K. (1988). Determinants of tumor blood flow: a review. *Cancer Res.*, **48**, 2641.

KAWAI, T., SATOMI, N., SATO, N. & 4 others (1987). Effects of tumour necrosis factor (TNF) on transplanted tumors induced by methylcholanthrene in mice. *Virchows Arch. B.*, **52**, 489.

KROSNICK, J.A., MULÉ, J.J., MCINTOSH, J.K. & ROSENBERG, S.A. (1989). Augmentation of antitumor efficacy by the combination of recombinant tumor necrosis factor and chemotherapeutic agents *in vivo*. *Cancer Res.*, **49**, 3729.

KUPER, C.F., BLOKSMA, N., BRUYNTJES, J.P. & HOFHUIS, F.M.A. (1988). Antitumor effects of endotoxin against solid murine Meth A tumors of different ages. Quantitative histology of the tumors and regional lymph nodes. *Virchows Arch. B.*, **56**, 51.

KUPER, C.F., BLOKSMA, N., HOFHUIS, F.M., BRUYNTJES, J.P. & WILLERS, J.M. (1982). Influence of adrenoceptor blockade on endotoxin-induced histopathological changes in murine Meth A sarcoma. *Int. J. Immunopharmacol.*, **4**, 49.

LEU, F.J. & DAMJANOV, I. (1988). Protease treatment combined with immunohistochemistry reveals heterogeneity of normal and neoplastic basement membranes. *J. Histochem. Cytochem.*, **36**, 213.

MURRAY, J.C., RANDHAWA, V. & DENEKAMP, J. (1987). The effects of mephalan and misonidazole on the vasculature of a murine sarcoma. *Br. J. Cancer*, **55**, 233.

NAKANO, K., ABE, S. & SOHMURA, Y. (1986). Recombinant human tumor necrosis factor – I. Cytotoxic activity *in vitro*. *Int. J. Immunopharmacol.*, **8**, 347.

NAWROTH, P., HANDLEY, D., MATSUEDA, G. & 4 others (1988). Tumor necrosis factor/cachectin-induced intravascular fibrin formation in Meth A sarcomas. *J. Exp. Med.*, **168**, 637.

NOWOTNY, A. (1985). Antitumor effects of endotoxins. In *Handbook of Endotoxin, Vol. 3: Cellular Biology of Endotoxin*, Berry, L.J. (ed.). p. 389. Elsevier Science: Amsterdam.

SEVICK, E.M. & JAIN, R.K. (1989). Viscous resistance to blood flow in solid tumors: effect of hematocrit on intratumoral blood viscosity. *Cancer Res.*, **49**, 3513.

SHEAR, M.J. & PERRAULT, A. (1943). Chemical treatment of tumors. IX. Reactions of mice with primary subcutaneous tumors to injection of hemorrhage-producing bacterial polysaccharide. *J. Natl Cancer Inst.*, **4**, 461.

TOZER, G.M., LEWIS, S., MICHALOWSKI, A. & ABER, V. (1990). The relationship between regional variations in blood flow and histology in a transplanted rat fibrosarcoma. *Br. J. Cancer*, **61**, 250.

VAN DE WIEL, P.A., BLOKSMA, N., KUPER, C.F., HOFHUIS, F.M. & WILLERS, J.M. (1989). Macroscopic and microscopic early effects of tumour necrosis factor on murine Meth A sarcoma, and relation to curative activity. *J. Pathol.*, **157**, 65.